# Geriatrics curricular reforms in oral health education: an interprofessional approach

**DOI:** 10.3389/fmed.2026.1776259

**Published:** 2026-06-16

**Authors:** Meenakshi Srinivasa Iyer, Raghunath Nagasundara Rao, Jaishankar H. P., Guranchal Chadha, Madhumita Raju Pillai

**Affiliations:** 1 Department of Prosthodontics and Crown & Bridge, JSS Dental College and Hospital, JSSAHER, Mysuru, India; 2 Department of Orthodontics and Dentofacial Orthopedics, JSS Dental College and Hospital, JSSAHER, Mysuru, India; 3 Department of Oral Medicine and Radiology, JSS Dental College and Hospital, JSSAHER, Mysuru, India

**Keywords:** curriculum reform, education, geriatric dentistry, global health, health policy, interprofessional, oral health, systemic health

## Abstract

The global shift toward an aging population requires a major change in oral health education. Traditional dental programs often fall short in meeting the complex needs of older patients. This can result in scattered care and poor health results. This paper advocates for incorporating Interprofessional Education (IPE) into geriatric oral health training. By combining evidence from the WHO-World Health Organization Framework for Action, research on the connection between oral and systemic health, and educational models from various countries, this paper presents a skills-based plan for changing the curriculum. It argues that interprofessional collaborative practice (IPCP) is essential not just for improving education but also for ensuring fair access to health services. The paper outlines a curriculum framework based on the “4Ms” of Age-Friendly Health Systems. It offers strategies for implementation, including high-fidelity simulation and task-shifting in places with limited resources. It also suggests solid evaluation methods based on the Kirkpatrick model which organizes impact into four levels: learners’ immediate reactions, gains in knowledge and skills, changes in behavior in real practice, and measurable effects on patient or system outcomes. Ultimately, it calls for a move from simply working together across disciplines to real collaboration to address the growing challenges of an aging population.

## Highlights

- Demographic Imperative: By 2050, the global geriatric population will double. This creates an urgent need for dentists skilled in managing multiple health issues and frailty, which single-discipline training cannot address.
- Systemic Nexus: Curricula must reflect the biological reality that oral diseases, such as periodontitis and dysphagia, are closely connected to systemic geriatric conditions like diabetes and pneumonia. This requires collaborative management.
- Competency Integration: Effective curricula blend IPEC Competencies–Values, Roles, Communication, and Teams–with geriatric-specific frameworks like the “4Ms” (Matters, Medication, Mentation, Mobility).
- Global Adaptability: High-income countries may emphasize nursing home rotations, while low- and middle-income countries should use task-shifting and mobile health units to provide interprofessional geriatric care in resource-limited settings.
- Assessment Rigor: Evaluation should go beyond student satisfaction, measured at Kirkpatrick Level 1. It must also assess changes in collaborative behavior (Level 3) and patient outcomes (Level 4) using validated tools like RIPLS.
- Leadership Requirement: Successful reform needs “institutional champions” to address logistical challenges, such as scheduling and silos, and requires policy support from accreditation bodies to ensure interprofessional exposure.
- Actionable Policy: Governments should require interprofessional education as a condition for licensure to break down academic silos.
- Ethical Obligation: Providing interprofessional care is a moral duty to offer holistic and dignified care to vulnerable members of society.

## 1 Introduction

### 1.1 The demographic imperative

By 2050, one in six individuals globally will be aged 65 years or older (1). In many industrialized nations, older adults already represent more than 20% of the total population, while emerging economies are experiencing accelerated aging within a shorter timeframe. The longevity revolution has shifted the disease burden from acute infectious illnesses toward chronic and degenerative conditions, including those affecting the oral cavity (2). Tooth retention into advanced age, although a public-health success, has created a new challenge: maintaining functional dentition and oral comfort in individuals with multiple comorbidities, cognitive decline, and functional limitations (3).

### 1.2 Oral health as a determinant of healthy aging

Oral health directly affects speech, nutrition, self-esteem, and social participation. Conditions such as dental caries, periodontitis, edentulism, reduced salivary secretion and xerostomia, and ill-fitting prostheses can reduce food intake and contribute to frailty and undernutrition (4). Poor oral hygiene has been linked to aspiration pneumonia, poor glycemic control in diabetes, and cardiovascular disease (5). Hence, oral health is both a biological and social determinant of health in later life. Addressing it requires coordinated care among dental and medical professionals, caregivers, and community support systems (6).

### 1.3 Fragmentation of geriatric oral healthcare

Despite the established oral–systemic connection, oral healthcare for older adults remains siloed. In many health systems, dental services are excluded from mainstream geriatric and primary-care pathways. Older adults in long-term care facilities often rely on non-dental staff for daily oral hygiene, yet those caregivers frequently lack training or institutional support (7). Studies reveal that fewer than 20% of nursing-home residents receive regular professional oral care (8). These gaps underscore the need for a workforce trained in collaborative geriatrics.

### 1.4 Why education reform is needed

Educational institutions are the foundation for preparing future practitioners. However, most dental and medical programs still provide minimal exposure to geriatric care (9). Surveys show that fewer than half of dental schools include dedicated geriatric dentistry rotations, and interprofessional experiences are rare, with evidence primarily limited to the UK, USA, and Canada (10). Curriculum overcrowding, insufficient faculty expertise, and limited community placements are recurring barriers. To close this gap, geriatrics must be embedded as a core theme across the educational continuum, supported by structured interprofessional learning.

## 2 Global demographic context and rationale

### 2.1 Aging trends and oral health burden

The world is aging at different speeds but with a consistent trajectory. The United Nations projects that the number of people aged 80 years and older will become threefold between 2020 and 2050 (11). This demographic shift corresponds with a rise in chronic oral conditions that require ongoing management rather than episodic treatment. Data obtained through the Global Burden of Disease Study (12) indicate that untreated dental caries and periodontal diseases remain among the most prevalent conditions worldwide, affecting older adults disproportionately.

### 2.2 Health-system implications

Aging populations generate increasing demand for integrated, cost-effective health services. Because oral health influences general health outcomes and healthcare expenditures, integrating oral health into geriatric care pathways is both clinically and economically justified (13). For example, improved oral hygiene in nursing-home residents has been associated with reduced rates of pneumonia and lower healthcare costs (14). Policymakers therefore advocate for including oral health in national healthy-aging frameworks (15).

### 2.3 Education as a lever for change

Workforce education is a sustainable mechanism for health-system transformation. The Institute of Medicine identified interprofessional education as essential for preparing practitioners capable of collaborative practice (16). Interprofessional training fosters understanding of overlapping roles and competencies, improves communication, and enhances care quality (17). Embedding geriatrics into this model ensures that oral health professionals learn to operate within complex, multidisciplinary teams caring for older adults with multimorbidity.

### 2.4 Ethical and social rationale

Equity in oral healthcare for older adults is a moral imperative. Ageism, social isolation, and financial constraints often marginalize this group (18). Curricular reforms that emphasize empathy, ethics, and advocacy can shift professional culture toward inclusive care. Interprofessional collaboration also democratizes expertise–allowing nurses, physicians, and caregivers to contribute meaningfully to maintaining oral health and dignity in aging (19).

### 2.5 Summary of rationale

**Table:** 

Rationale dimension	Key argument	Supporting evidence
Demographic	Rapid increase in ≥65 population	(1, 11) Global aging statistics
Clinical	Oral–systemic linkages	(4–6) Diabetes, cardiovascular, and pneumonia risks
Educational	Insufficient geriatric and IPE exposure	(9–10, 16–17) Surveys of dental curricula
Economic	Preventive oral care reduces cost burden	(13–14) Health-system studies
Ethical	Older adults’ right to dignified oral health	(18–19) WHO Healthy Aging principles

## 3 Evidence supporting interprofessional education in geriatrics

### 3.1 Global population aging and implications for oral health care delivery

The demographic shift detailed in the Section “2.1 Aging trends and oral health burden” necessitates a transition from traditional restorative models to an integrated geriatric care management. As this “silver tsunami” reaches its peak, with nearly 80% of older adults residing in LMICs by 2025, the challenge evolves from a clinical one into a systemic “syndemic” where oral disease compounds to general frailty (20, 21).

This change fundamentally affects how oral health care is delivered. Older adults face a heavy burden of oral disease, which includes high rates of periodontitis, root caries, tooth loss, and oral cancer. Additionally, the idea of “frailty” adds a new layer to care that goes beyond the technical skills of restorative dentistry. In wealthy countries like Japan and Sweden, the “8020” movement (keeping 20 teeth by age 80) has been successful, but it has also led to a new challenge: patients with cognitive decline or physical impairment often have “heavily restored” teeth. These patients need complex care that a dentist cannot provide alone. Meanwhile, in countries like India and Brazil, the sharp increase in non-communicable diseases (NCDs) coincides with a significant unmet dental need. Delivery systems must integrate oral health into general medicine to prevent oral disease from accelerating physical frailty (22–24).

### 3.2 Burden of geriatric oral diseases and links to systemic health outcomes

The biological plausibility linking oral health to systemic status provides the strongest curricular argument for Interprofessional Education (IPE). The mouth is not an isolated ecosystem; it is the portal to the body.

- Aspiration pneumonia: this is a leading cause of mortality in institutionalized elders. It is directly linked to poor oral hygiene and dysphagia. Pathogens such as *Porphyromonas gingivalis* and *Staphylococcus aureus* from the oral cavity are aspirated into the lungs, causing infection. Systematic reviews have demonstrated that professional oral care interventions can significantly reduce the incidence of fatal pneumonia in nursing home residents, but this requires coordination between nursing staff (daily care) and dental professionals (professional debridement) (25, 26).
- Diabetes mellitus: the bidirectional relationship between periodontal disease and diabetes is well-established. Periodontal inflammation adversely affects glycemic control, while hyperglycemia impairs wound healing in the oral cavity. Managing a geriatric diabetic patient requires a dental student to understand HbA1c targets (traditionally the domain of medicine) and a medical student to recognize periodontal inflammation (27).
- Alzheimer’s disease: emerging evidence suggests a potential link between periodontal pathogens and Alzheimer’s disease, positing that chronic oral inflammation contributes to the systemic inflammatory burden associated with neurodegeneration (28).

These connections underscore that the *siloed praxis* of current education–where “the teeth belong to the dentist, and the body belongs to the physician”–is clinically dangerous (29).

### 3.3 Conceptual and policy foundations of interprofessional education

The theoretical foundation for this reform is the World Health Organization Framework for Action on Interprofessional Education and Collaborative Practice from 2010. The WHO says that synergistic practice occurs when multiple health workers among various professional backgrounds gives comprehensive services by working with patients, their families, caregivers, and communities to provide the highest quality of care in different settings. Importantly, this framework asserts that interprofessional education is the necessary precursor to collaborative practice (30).

Supporting this are the CAIPE (Center for the Advancement of Interprofessional Education) principles, which define interprofessional education as when two or more professions learn with, from, and about one another to improve collaboration and the quality of care. This definition sets apart true interprofessional education from multiprofessional education, where students from different professions sit together in a lecture hall without interaction (31).

### 3.4 Global empirical evidence supporting IPE in geriatrics

Evidence suggests that IPE interventions in geriatrics yield measurable improvements, though the landscape is uneven.

- Japan: In acute care settings, interprofessional oral health support programs involving dentists, nurses, and speech therapists significantly reduced tongue microbial counts and improved caregiver motivation (32).
- United States: Curricula aligned with the Interprofessional Education Collaborative (IPEC) have demonstrated that dental students participating in interdisciplinary geriatric rotations exhibit higher empathy and readiness for complex case management (33) (Table 1).
- Brazil: Models in the Unified Health System (SUS) demonstrate how integrating dental teams into primary care centers (Family Health Strategy) can improve diabetes management in the elderly through coordinated oral care (34) (Table 2).

**TABLE 1:** Global interprofessional competency framework for geriatric oral health.

Domain	Competency objective	Specific geriatric application
Values/ethics	Work alongside people from other professions to promote respect and shared principles.	Recognize the ethical imperative of oral care in palliative and end-of-life contexts (37). Respect the autonomy of elders with cognitive decline.
Roles/responsibilities	Use knowledge of your role and other professionals to properly assess and address patient care needs	Dentistry: Manage odontogenic pain/infection (38). Nursing: Daily oral hygiene maintenance. Pharmacy: Management of xerostomic medications. Medicine: Clearing patients for surgical interventions.
Interprofessional communication	Engage in responsive and responsible communication with patients, families, communities, and professionals in healthcare and related disciplines.	Utilize “Age-Friendly” communication techniques (e.g., addressing sensory deficits). Effective hand-off of oral health status during hospital discharge or admission to long-term care (39).
Teams and teamwork	Apply teamwork and relationship building skills to perform well in different team roles.	Participate in multidisciplinary geriatric case conferences. Lead or support the “Oral Health” component of the Comprehensive Geriatric Assessment (CGA) (40).

**TABLE 2:** Comparative implementation strategies.

Strategy	HIC context (e.g., USA, Sweden)	LMIC context (e.g., India, Vietnam, Brazil)
Clinical setting	University-affiliated nursing homes/LTCFs.	Community Health Centers (CHC) and mobile dental units visiting rural villages (50).
Workforce model	Dentist-led multidisciplinary teams.	Task-Shifting: Utilizing Dental Therapists, Anganwadi workers (India), or Community Health Workers to perform screening and basic education, supervised remotely (51).
Technology	Electronic Health Records (EHR) with integrated dental tabs.	Tele-dentistry applications (WhatsApp-based triage) connecting rural workers to urban dental specialists (52).
Simulation	High-fidelity geriatric mannequins.	Role-play with standardized patients (actors) or peer-to-peer simulation using age-simulation suits (53).

However, barriers remain. A systematic review of dental participation in geriatric IPE found that while students often report high satisfaction, data on long-term behavioral change is scarce. In India, the lack of interprofessional referral mechanisms remains a primary barrier, highlighting the cost of not educating for collaboration (35, 36).

## 4 Curriculum framework and design principles

### 4.1 Competency-based education for geriatric oral health

Curricular reform must move beyond “time-in-seat” to demonstrate competence. We propose a hybrid framework that integrates the IPEC Core Competencies (2023) accompanied by Age-Friendly Health Systems “4Ms” Framework (What Matters, Medication, Mentation, Mobility).

### 4.2 Vertical and horizontal integration of geriatrics

Integration must occur at two levels to be effective:

- Horizontal integration (pre-clinical): basic science courses (e.g., physiology of aging) should be co-taught. Dental and medical students should jointly learn the mechanism of dysphagia, understanding both the muscular pathology (medicine/speech pathology) and the implications of tooth loss/prosthetics (dentistry) (41).
- Vertical integration (clinical): as students progress, IPE must shift to clinical settings. Third-year dental students should rotate through geriatric medicine ward, while medical residents should rotate through geriatric dental clinics to understand the complexity of delivering care to frail elders. This reciprocal rotation model desensitizes medical students to the oral cavity and exposes dental students to complex medical management (42).

### 4.3 Application of adult learning theory and experiential learning

According to this theory, also known as Andragogy, given by Malcolm Knowles, suggests that professional students learn best when instruction focuses on real problems and can be applied right away. Scenario-Based Peer Learning (SBPL) works well in this context. For instance, a European study used SBPL where dental students analyzed cases with other health professionals. This approach led to significantly higher outcome on the Readiness for Interprofessional Learning Scale (RIPLS). Reflection plays a crucial role; students need to discuss not only the clinical results but also the team dynamics that affected those results. By using Donald Schön’s idea of “reflection-in-action,” students learn to manage the complex and uncertain aspects of practicing geriatric care (43–45).

### 4.4 Alignment with global accreditation standards

Curricula must align with accreditation bodies to ensure sustainability.

- USA: The Commission on Dental Accreditation Standard 2–25 requires graduates to be capable in communicating and getting together with other members of the healthcare team (46).
- Europe: The Association for Dental Education in Europe (ADEE) includes “Social Accountability” and “Team Leadership” as core domains in its graduating European Dentist profile (47).
- Global: In regions without specific IPE mandates, aligning with the FDI World Dental Federation’s Vision 2030–which prioritizes integration into general health–provides a strategic policy lever.

## 5 Implementation roadmap

### 5.1 Institutional readiness and leadership commitment

Successful implementation requires a change in institutional culture. Leaders must identify “champions” within both the dental faculty and partner schools, such as Nursing, Medicine, and Social Work. In many institutions, especially in low and middle income countries, the separation of dental schools from general university hospitals creates a barrier. Leadership must establish “virtual” or “rotational” connections when physical co-location is not possible. The idea of “institutional isomorphism” suggests that dental schools will eventually adopt the collaborative structures of medical schools if accreditation and funding bodies provide enough incentives.

### 5.2 Stakeholder engagement across health professions

- Nursing: The most critical partner for geriatric oral health. Nurses deliver daily care in Long-Term Care Facilities (LTCF). Curricula must include joint workshops that empower the nursing staff to:
- Perform comprehensive daily oral hygiene and denture care.
- Identify clinical signs of oral candidiasis and other mucosal pathologies
- Manage the symptoms of xerostomia (dry mouth and reduced saliva secretion) to improve patient function.
- Implement standardized screening instruments, such as the Revised Oral Assessment Guide (ROAG), to identify patients requiring urgent professional dental intervention (48).
- Pharmacy: Polypharmacy is ubiquitous in geriatrics. Joint seminars on drug-induced xerostomia, the subsequent high risk of rampant caries, and osteonecrosis of the jaw facilitate mutual understanding (49).
- Social Work: Assessing the financial and social barriers to dental access for the elderly is a core competency often missing in dental education. Social work students can teach dental students how to navigate social safety nets for their patients.

### 5.3 Teaching–learning strategies: case studies

#### 5.3.1 Simulation-based interprofessional education

Simulation lets students practice critical situations in a safe setting.

For example, in a “Geriatric Trauma” simulation, a dental student, nursing student, and medical resident work together to care for a patient who has fallen, fractured a hip, and lost teeth. The team needs to focus on the airway, control pain, and coordinate stabilization.

#### 5.3.2 Community-based clinical rotations (global contrast)

Implementation strategies must be resource appropriate. We distinguish between Higher Income Countries (HICs) and Low to Middle Income Countries (LMICs).

#### 5.3.3 The “ward model” (Sweden)

The Karolinska Institute has developed the “clinical ward” model. In this program, students from dental sciences (including both dental and dental hygiene students), medicine, nursing, and physiotherapy manage a ward of real patients under supervision for 2 weeks. This hands-on experience helps students understand their roles better (54).

### 5.4 Resource planning, scalability, and sustainability

Sustainability depends on incorporating oral health into existing national health missions. For example, Vietnam has integrated the “Basic Package of Oral Care” (BPOC) into general health training. This approach creates a model where non-dentists can help with the oral health needs of the elderly. In India, the large network of Anganwadi workers conducts basic oral screenings for older adults, creating a sustainable workforce (30).

## 6 Evaluation and quality assurance

### 6.1 Assessment of learner outcomes

Evaluation must focus on how well students gain interprofessional skills, not just dental knowledge.

Psychometric Scales: RIPLS: The Readiness for Interprofessional Learning Scale is the standard tool for assessing student attitudes before and after interventions. Another tool is the Interdisciplinary Education Perception Scale (IEPS), which measures professional perception (55).

OSCEs: Objective Structured Clinical Examinations should have “Interprofessional Stations.” For instance, one station could require a dental student to explain a patient’s inability to chew to a dietitian and work together to create a nutritional plan (56).

### 6.2 Program evaluation frameworks

The Kirkpatrick Model is recommended for its four-level approach to evaluate the education programmes:

1. Level 1 (Reaction): the focus is on student satisfaction with the IPE module and perceived relevance.
2. Level 2 (Learning): change in knowledge skills, and attitudes are captured using geriatric knowledge tests and RIPLS.
3. Level 3 (Behavior): Change in referral patterns or communication style in clinics (assessed via chart audits) (57).
4. Level 4 (Results): (The ultimate goal) Improved patient health outcomes (e.g., reduced aspiration pneumonia rates in partner nursing homes).

### 6.3 Accreditation benchmarks and best practices

Programs should use international standards for comparison. The Interprofessional Health Education Certification (IHEC) is becoming a global standard. Continuous Quality Improvement (CQI) methods must be set up, using feedback from community advisory boards made up of elderly patients and caregivers. This feedback will help keep the curriculum aligned with real-world needs.

### 6.4 Challenges and mitigation strategies

- Logistics (“Schedule Tetris”): Aligning calendars of different schools is the most cited barrier. Mitigation: Use “Interprofessional University Days” where all classes are suspended for joint activities or utilize asynchronous online modules.
- Tribalism: Professional stereotypes can hinder collaboration. Mitigation: Early exposure–introduce IPE in the first year before stereotypes harden (“socialization into the profession” vs. “socialization into the team”).

## 7 Conclusion and recommendations

### 7.1 Synthesis of key arguments

The separation of oral health from general health education is an outdated practice that puts the aging population at risk. The evidence is clear: geriatric oral health is complicated, systemic, and social. It cannot be handled alone. Interprofessional education (IPE) is the only effective way to prepare a workforce ready to face the challenges of the “silver tsunami.”

### 7.2 Strategic recommendations

1. For Educators: Go beyond traditional lectures. Use “service-learning” so students learn to work together by solving real problems in underserved geriatric communities.
2. For Academic Leaders: Break down administrative barriers. Coordinate calendars between health schools to encourage joint learning. Create “joint appointments” for faculty who connect dentistry and medicine.
3. For Policymakers: Require interprofessional education (IPE) for licensure and accreditation. In low- and middle-income countries, policies should support “task-shifting” laws to enable teamwork between dentists and community health workers.
4. For Global Health: Include oral health indicators in general geriatric health surveys to collect data that can influence policy.

### 7.3 Implications for global workforce development

The “collaborative practice-ready workforce” is not a luxury for wealthy nations; it is a necessity for everyone. In LMICs, where specialists are few, a dentist’s ability to train a nurse, or a community health worker’s ability to refer to a dentist, can mean the difference between quality care and neglect.

### 7.4 Priority areas for future research

Future research should focus on longitudinal studies that track dental graduates into practice to determine if IPE exposure changes how they treat geriatric patients. Cost-benefit analyses of IPE-based geriatric care in low-resource settings are also important.

## Data Availability

The original contributions presented in this study are included in this article/supplementary material, further inquiries can be directed to the corresponding author.
